# Low Level of Dietary Organic Trace Minerals Improved Egg Quality and Modulated the Status of Eggshell Gland and Intestinal Microflora of Laying Hens During the Late Production Stage

**DOI:** 10.3389/fvets.2022.920418

**Published:** 2022-06-29

**Authors:** Yuanyang Dong, Keke Zhang, Miaomiao Han, Zhiqiang Miao, Ci Liu, Jianhui Li

**Affiliations:** Department of Livestock Production, College of Animal Science, Shanxi Agricultural University, Taigu, China

**Keywords:** egg quality, eggshell gland, laying hen, mineral deposition, organic trace mineral element

## Abstract

This study aimed to investigate the effects of dietary organic trace minerals on egg quality and intestinal microflora of laying hens during the late production stage. In total, 1,080 Jinghong-1 laying hens aged 57 weeks were randomly assigned to five treatment groups: CON, basal diet containing about 6, 29, 49, and 308 mg·kg^−1^ of Cu, Mn, Zn, and Fe; IT100, basal diet supplemented with 10, 80, 80, and 60 mg·kg^−1^ of Cu, Mn, Zn, and Fe (each as inorganic sulfates), respectively; OT20, basal diet supplemented with 2, 16, 16, and 12 mg·kg^−1^ of Cu, Mn, Zn, and Fe (each as organic trace minerals chelated with lysine and methionine in the ratio of 2:1 amino acid: organic trace minerals), respectively; OT30, basal diet supplemented with 3, 24, 24, and 18 mg·kg^−1^ of organic Cu, Mn, Zn, and Fe, respectively; and OT50, basal diet supplemented with 5, 40, 40, and 30 mg·kg^−1^ of organic Cu, Mn, Zn, and Fe, respectively. Overall, OT20, OT30, and OT50 had equal or higher potential to promote Cu, Mn, Zn, and Fe deposition in egg yolks compared with IT100. In addition, OT50 enhanced the eggshell breaking strength and the antioxidant status of the eggshell gland. Cecal microbiota, including *Barnesiellaceae* and *Clostridia*, were significantly decreased in IT100- and OT50-treated hens compared with the CON group. *Clostridia* UCG-014 was negatively correlated with eggshell weight and OCX-32. In conclusion, reduced supplementation of organic trace minerals can improve the eggshell quality and trace mineral deposition, possibly by modulating genes involved in the eggshell formation in the eggshell gland and by controling of the potentially harmful bacteria *Barnesiellaceae* and *Clostridiales* in the cecum. Inorganic trace minerals may be effectively replaced by low level of complex organic trace minerals in laying hens during the late production stage.

## Introduction

Eggshells are complex structures that protect the inner egg content; however, the eggshell percentage, breaking strength, and egg color (redness) are reduced with aging in laying hens at 30–81 weeks of age (1). A high percentage of cracked eggs due to thin eggshells is a common problem during the late production stage of laying hens (2). Eggshell strength is a major issue during egg collection, transportation, storage, and processing, causing financial losses to the egg industry (1). The loss from damaged eggshells accounts 8–11% of total egg loss (3). Hence, the improvement of egg quality is of great importance for the poultry industry.

Previous studies showed that trace mineral nutrition improves eggshell quality. Microelements such as Zn, Mn, and Cu, as cofactors of certain enzymes could affect the eggshell quality through influencing the mineralization and microstructure of eggshells (4). For instance, Zn as a cofactor of carbonic anhydrase which catalyzes carbon dioxide into bicarbonate ions, is closely linked to eggshell formation by affecting the crystal and texture morphologies of the shell (3); Mn is a cofactor of metalloenzymes responsible for carbonate and mucopolysaccharide synthesis and plays an important role in eggshell formation (5); Fe increases the egg production along with increasing blood hemoglobin without influencing the eggshell color or egg components, compared with Fe depletion pretreatment group (6); and Cu functions as an enzyme constituent (i.e., lysyl oxidase, a cuproenzyme involved in conversion of lysine to cross-linked desmosine and isodesmosine), and the deficiency of Cu resulted in abnormal eggshell formation (7). Interaction with dietary constituents and interference between different trace minerals limit the absorption of inorganic trace minerals (8). For instance, Zn-methionine has a higher bioavailability than inorganic Zn (i.e., Zn sulfate), because it is more stable and resists interference from other ligands in the gastrointestinal tract (3). Moreover, aging also resulted in physiological degeneration of the gastrointestinal tract, which decreases the absorption and bioavailability of micronutrients (9). The gut microbiota also affects the mineral metabolism by directly influencing mineral absorption in the gastrointestinal tract and by producing enzymes such as phytase, to help release mineral from diet (10). The relationship between trace minerals and microbiome is bidirectional. Trace minerals can alter the composition, function of intestinal microbiota, and compartmentalize metabolic inflammation (11). Previous studies mainly focused on a single organic trace mineral (i.e., Fe-glycine or Zn-methionine) without investigating the antagonistic effects of trace minerals on absorption and utilization. For example, increased concentration of Zn, Fe in diet could reduce the availability of Cu (7). In addition, high level of dietary inorganic trace minerals (ITM) is often applied to meet the requirements of birds, leading to more mineral excretion into the environment than organic trace minerals (12).

Eggshell is formed in the shell gland of laying hens and genes related to organic anion transport, secretion and signal release were regulated during eggshell formation (13). Several genes have been reported to be associated with the eggshell biomechanical properties and contributed to the eggshell formation. For example, single nucleotide polymorphisms (SNPs) in *ovocleidin-116 (OC-116)* gene was related with eggshell modulus, thickness; *ovocleidin-17 (OC-17)* was predicted to catalyze the transformation of amorphous calcium carbonate into calcite, the main component of mineralized shell; *ovocalyxin-32 (OCX-32)* SNPs were reported to be significantly associated with mammillary layer thickness; *osteopontin (OPN)* was associated with eggshell fracture toughness (14). We have found that compared with the inorganic trace mineral, reduced supplementation of complexed organic trace minerals showed equal or increased mineral deposition in the eggshell, and reduced emission to the environment without negative effects on laying performance (15). As improve decreased mineral deposition in the eggshell, the eggshell matrix protein may be also upregulated, which contributed to the eggshell quality. Thus, the present study aimed to investigate the effects of dietary complex organic trace minerals on eggshell quality, intestinal microflora, and status of eggshell gland of laying hens during the late production stage.

## Materials and Methods

### Bird and Management

A total of 1,080 laying hens (Jinghong-1 strain) at 57 weeks of age were randomly assigned to five treatment groups (CON, basal diet; IT100, basal diet supplemented with 10 mg·kg^−1^ Cu, 80 mg·kg^−1^ Mn, 80 mg·kg^−1^ Zn, and 60 mg·kg^−1^ Fe as inorganic sulfates; OT20, basal diet supplemented with 2 mg·kg^−1^ Cu, 16 mg·kg^−1^ Mn, 16 mg·kg^−1^ Zn, and 12 mg·kg^−1^ Fe as organic trace minerals; OT30, basal diet supplemented with 3 mg·kg^−1^ Cu, 24 mg·kg^−1^ Mn, 24 mg·kg^−1^ Zn, and 18 mg·kg^−1^ Fe as organic trace minerals; and OT50, basal diet supplemented with 5 mg·kg^−1^ Cu, 40 mg·kg^−1^ Mn, 40 mg·kg^−1^ Zn, and 30 mg·kg^−1^ Fe as organic trace minerals) with six replications and 36 layers (12 cages per replicate and three layers per cage). The basal diet (Table 1) was formulated to meet the national Nutrient Requirements of Laying hens recommended by the NY/T33-2004(2004) released by the Chinese Academy of Agricultural Sciences. The organic trace elements were provided as amino acid chelates with amino acids and metal elements chelated at 2:1 moles, containing 1.48% lysine and 0.13% methionine and the purity of Cu, Zn, Mn, Fe ≥ 99%, which was purchased from Changning DeBon Biotech (Hunan, China). The analyzed levels of Cu, Zn, Mn, Fe in each experiments were shown below: CON, 38.21 mg·kg^−1^ Cu, 35.00 mg·kg^−1^ Mn, 34.19 mg·kg^−1^ Zn, 66.09 mg·kg^−1^ Fe; IT100, 99.43 mg·kg^−1^ Cu, 120.45 mg·kg^−1^ Mn, 116.20 mg·kg^−1^ Zn, and 130.09 mg·kg^−1^ Fe; OT20, 50.16 mg·kg^−1^ Cu, 52.55 mg·kg^−1^ Mn, 51.40 mg·kg^−1^ Zn, and 80.40 mg·kg^−1^ Fe; OT30, 57.09 mg·kg^−1^ Cu, 63.65 mg·kg^−1^ Mn, 60.05 mg·kg^−1^ Zn, and 88.90 mg·kg^−1^ Fe as organic trace minerals; and OT50, 68.83 mg·kg^−1^ Cu, 82.24 mg·kg^−1^ Mn, 75.00 mg·kg^−1^ Zn, and 100.06 mg·kg^−1^ Fe. The birds were housed in cages at 25°C with a photoperiod of 16 h throughout the experimental period (acclimation, 2 weeks; experimental treatment, 8 weeks). Food and water were available *ad libitum*. The present study was approved by the Animal Health and Care Committee of the Shanxi Agricultural University (Shanxi, China) and conducted with the Guidelines for the Experimental Animal Welfare of Ministry of Science Technology of China.

**Table 1:** Ingredients and nutrient compositions of the basal diets.

**Ingredients, %**		**Nutrient composition, %**	
Corn	66.21	ME (MJ/kg)	11.31
Soybean meal	21.00	CP, %	15.36
Wheat bran	2.00	Lysine, %	0.80
Limestone	8.00	Methionine, %	0.35
Dicalcium phosphate	2.00	Methionine + Cysteine, %	0.62
Salt	0.30	Calcium^b^, %	3.61
DL-Methionine (98%)	0.10	Total phosphorus^b^, %	0.49
L-Lysine (78%)	0.10	Cu (mg/kg)	38.21
Choline chloride (50%)	0.10	Zn (mg/kg)	34.19
Premix^a^	0.19	Mn (mg/kg)	35.00
Total	100.00	Fe (mg/kg)	66.09

a *The vitamin premix provided per kg diet: vitamin A, 9,500 IU; D_3_ 3,500 IU; vitamin E 20 IU; vitamin K_3_ 2 mg; vitamin B_1_ 5 mg; vitamin B_2_ 7 mg; vitamin B_6_ 5 mg; vitamin B_12_ 0.02 mg; folic acid 1 mg; biotin 0.2 mg; niacin 45 mg; calcium pantothenate 10 mg; I 1 mg/kg; Se 0.3 mg/kg*.

b *Calcium, total phosphorus and Cu, Zn, Mn, and Fe are measured values, others are based on calculated values*.

### Sample Collection and Measurements

#### Sample Collection

Feed intake, egg weight, egg mass, feed conversion ratio (g of feed to g of egg) and mortality were calculated at the end of the trail in 8th week. The egg was collected and weighed by replicate at 1,400 p.m. every day. Feed intake was recorded every 2 weeks. At the end of week 8 of the experimental period, six birds from each treatment were randomly selected and euthanized. The eggshell gland tissue and liver were obtained under sterile conditions and stored at −80°C for further analysis.

#### Deposition of Trace Minerals in Egg Yolk and Liver

Mixed egg yolks from the same replicate and the liver were frozen, vacuum-dried, and stored at −80°C. Egg yolk, live samples (0.2 g each) were placed into digestion tubes and mixed with 6 ml of HNO_3_ and 2 ml of H_2_O_2_ at 25°C overnight. The mixed samples were digested using a MARS6 microwave digestion instrument (CEM Corporation, Matthews, NC, USA) at 120°C for 5 min, 150°C for 5 min, and 180°C for 30 min. The samples were placed in an EHD-24 acid drive instrument (Beijing Donghang Sci-Tech Instrument, Beijing, China) until 1 ml of liquid was left. The digestion in the tubes was diluted and the volume adjusted to 50 mL with double distilled water. The contents of Cu, Mn, Zn, and Fe in the egg yolk and liver were determined using an iCAP Q inductively coupled plasma mass spectrometer (Thermo-Fisher Scientific, Waltham MA, USA), according to the method of Gheisari et al. (7).

#### Egg Quality

At the end of weeks 6 and 8 of the experimental period, 10 eggs from each replicate were randomly collected for egg yolk quality evaluation. The egg yolk quality consisted of egg yolk color, weight, and egg yolk ratio. The egg yolk color was evaluated using an EA-01 egg analyzer (Israel Orka Food Technology) with RYCF units (Roche Yolk Color Fan units). The egg yolk was weighed to calculate the egg yolk ratio (egg yolk weight/whole egg weight × 100).

#### Antioxidant Status of Eggshell Gland

To determine the antioxidant activity of the eggshell gland, the tissue was homogenized in pre-cold 0.9% saline and centrifuged at 3,000 rpm and 4°C for 10 min. The supernatant was preserved at −20°C for further analysis. The activities of total antioxidant capacity (T-AOC), catalase (CAT), total superoxide dismutase (T-SOD), and malondialdehyde (MDA) were analyzed using commercial kits using commercial colorimetric kits (Nanjing Jiancheng Bioengineering Institute, Nanjing, China), according to the manufacturer's instructions. The values of T-AOC, CAT, and T-SOD in the eggshell gland tissue were expressed in U mg^−1^ of protein, while MDA was expressed in nmol mg^−1^ of protein. Protein concentrations in the supernatant were measured using a BCA Protein Assay kit (Nanjing Jiancheng Bioengineering Institute, Nanjing, China).

#### Gene Expression Associated With Eggshell Quality

The total RNA of the eggshell gland tissue was extracted using the Eastep Super Total RNA Extraction Kit (Promega, Madison, WA, USA). The RNA quality and quantity were examined using a Nanodrop spectrophotometer (Thermo-Fisher Scientific) at 260 nm and 280 nm. Total RNA was reverse transcribed using the PrimeScrip RT kit with gDNA Eraser Perfect Real-Time (Takara Biomedical Technology, Beijing, China). The transcription levels of *ovocleidin-116 (OC-116), ovocalyxin-32 (OCX-32), osteopontin (OPN), aminolevulinic acid synthase (ALAS)*, and *ferrochelatase (FECH)* were determined using the SYBR Premix Ex Taq Ti RNaseH Plus (Takara Biomedical Technology), according to the manufacturer's protocol. β-actin was used as a housekeeping gene, and the 2^−Δ*ΔCt*^ method was used to calculate the relative gene expression levels. The primers used in this study are presented in Table 2.

**Table 2:** Primer used for Real-time PCR of eggshell gland.

**Gene**	**Forward primer**	**Reverse primer**	**Accession number**
OC-116	ACTGGCTGGGTAAGGTGGATGG	CTTGCTGGAGTGATGTGGCTGTG	AF148716.3
OCX-32	ACAGAGCACACGGGTTACTTATTGG	TTAAACGCAACAGCATTGTCCTTCC	AJ307060.2
OC-17	GCAATGCCTTCGTCTGCAAAG	CTGCTGTGGGTCCGTTTATTG	KF835610.1
OPN	GAGCGTAGAGAACGACAGCC	CGCTCTCTAGCGTCTGGTTG	NM_204535.4
ALAS	TACGGCGGACCCACACATACC	GAGCACCACAACTCCACGGATG	M24367.1
FECH	GACCTCATGACGCTTCCAGC	GCCGTCCACTTCTTGATGGG	NM_204196.1
β-actin	CCCAAAGCCAACAGAGAGAAGATGAC	GTAACACCATCACCAGAGTCCATCAC	L08165.1

#### Cecal Microbiota Populations

The cecum was removed from the birds and the chyme was placed into a sterile tube, snap-frozen in liquid nitrogen, and stored at −80°C for further analysis. Multiplexed 16S rDNA libraries were prepared using standard *16S* metagenomic sequencing library protocols from Illumina (San Diego, CA, USA) with the V3–V4 region of *16S* rDNA for target amplification. Illumina MiSeq was used to perform paired-end reads (300 bp) to generate ~200,000 sequences per sample. Subsequent analysis was performed in QllME using Barcode to merge paired-end reads (mismatch, minimum score < 20). Additional quality filter steps were applied to exclude short reads (<230 bp) and chimeras. Samples producing < 35,000 reads were excluded to ensure high coverage of all samples. OUT-based enumeration of taxa with a similarity level of 97% and each operational taxonomic units (OTU) were annotated using the SILVA 16S rRNA database (bacteria and archaea; https://www.arb-silva.de). The fastq files generated by Illumina sequencing were uploaded to the NCBI BioProject database (BioProject ID, PRJNA738271; Submission ID, SUB9857288).

#### Statistical Analysis

Differences among treatments were identified using a one-way analysis of variance in conjunction with Duncan's test for multiple comparisons. Significance was set at *p* < 0.05. Spearman's rank correlation analysis was applied to identify associations between the relative abundance of cecal bacteria and indicators of the eggshell gland and eggshell quality. All analyses were carried out with SPSS 20.0 (IBM, Armonk, NY, USA).

## Results

### Deposition of Trace Minerals in Liver and Egg Yolk

OT30 and OT50 positively affected the deposition of trace minerals in the egg yolk (Table 3). IT100, OT20, OT30, and OT50 significantly increased the deposition of Cu, Mn, Zn, and Fe in the liver compared with CON; however, the effects of OT50 and IT100 did not differ significantly. Moreover, deposition of trace minerals in egg yolk did not differ significantly between CON and IT100, but both OT30 and OT50 showed a significant positive effect (*p* < 0.01).

**Table 3:** Effects reduced substitution of organic trace minerals on deposition of Fe, Cu, Mn, and Zn of liver, egg yolk at 8th week of experimental period with hen layer at the age of 67 weeks (μg/g, dry matter basis).

**Item**		**Treatment** ^a^						
		**CON**	**IT 100**	**OT 20**	**OT 30**	**OT 50**	**SEM** ^b^	***P*** **-values**
Liver	Cu	4.15^D^	5.04^A^	4.50^C^	4.78^B^	5.03^A^	0.053	<0.001
	Mn	4.28^C^	4.91^A^	4.64^B^	4.88^A^	4.90^A^	0.037	<0.001
	Zn	50.14^D^	63.14^A^	56.98^C^	58.83^B^	61.46^A^	0.679	<0.001
	Fe	114.92^C^	143.05^A^	133.00^B^	137.07^AB^	143.38^A^	1.669	<0.001
Egg yolk	Cu	1.20^B^	1.26^B^	1.22^B^	1.38^A^	1.36^A^	0.010	<0.001
	Mn	1.47^B^	1.54^B^	1.48^B^	1.69^A^	1.69^A^	0.016	<0.001
	Zn	8.54^B^	8.81^B^	8.61^B^	9.87^A^	9.88^A^	0.118	<0.001
	Fe	70.94^B^	72.27^B^	71.32^B^	82.45^A^	82.97^A^	0.782	<0.001

a *CON, basal diet without supplement of trace minerals; IT 100, basal diet with supplement of Fe (60 ppm), Cu (10 ppm), Mn (80 ppm) and Zn (80 ppm); OT 20, basal diet+20% IT 100 trace minerals; OT 30, basal diet+30% IT 100 trace minerals; OT 50, basal diet+50% IT 100 trace minerals*.

b *SEM, Standard error of the mean*.

*Means within a row lacking a common superscript differ (P < 0.05)*.

### Egg Yolk Quality

The egg yolk quality is summarized in Table 4. Egg yolk color and weight were not affected at week 6 or 8 and egg yolk ratio was not affected at 8 weeks of feeding. Nevertheless, IT100, and OT50 treatments significantly increased the ratio of egg yolk at week 6 of the experimental period (*p* < 0.01).

**Table 4:** Effects of inorganic and reduced substitution of organic trace minerals on egg yolk quality at the 6, 8 week of the experiment with hen layer at the age of 67 weeks.

**Item**	**Treatment** ^a^						
	**CON**	**IT 100**	**OT 20**	**OT 30**	**OT 50**	**SEM** ^b^	***P*** **-values**
**6 weeks**							
Egg yolk color^c^	5.76	5.87	5.47	5.93	5.67	0.086	0.468
Egg yolk weight (g)	16.66	17.48	17.25	17.42	17.36	0.108	0.112
Egg yolk ratio^d^ (%)	25.28^B^	26.42^A^	26.21^AB^	26.27^AB^	26.49^A^	0.134	0.029
**8 weeks**							
Egg yolk color	6.20	6.03	6.13	6.27	6.43	0.097	0.775
Egg yolk weight (g)	17.29	18.01	17.72	17.92	18.03	0.094	0.073
Egg yolk ratio (%)	26.16	26.88	26.40	26.76	26.87	0.119	0.220

a *CON, basal diet without supplementation of trace minerals; IT 100, basal diet supplemented with Fe (60 ppm), Cu (10 ppm), Mn (80 ppm), and Zn (80 ppm); OT 20, basal diet+20% IT 100 trace minerals; OT 30, basal diet+30% IT 100 trace minerals; OT 50, basal diet+50% IT 100 trace minerals (n = 6)*.

b *SEM, Standard error of the mean*.

c *Egg yolk color was evaluated by “Roche Yolk Color Fan (RYCF)” and measured in RYCF units*.

d *Egg yolk ratio was calculated as egg yolk weight/whole egg weight × 100*.

*Means within a row lacking a common superscript differ significantly (P < 0.05)*.

### Antioxidant Status of Eggshell Gland

CAT and T-SOD activities in the eggshell gland were increased, whereas MDA content was decreased in IT100, OT20, OT30, and OT50 groups compared with those in CON (Table 5). In addition, only IT100, OT30, and OT50 treatments increased T-AOC activity in the eggshell gland (*p* < 0.01).

**Table 5:** Effects of inorganic and reduced substitution of organic trace minerals on antioxidant status of eggshell gland at the 8th week of the experiment with hen layer at the age of 67 weeks.

**Item** ^c^	**Treatment** ^a^						
	**CON**	**IT 100**	**OT 20**	**OT 30**	**OT 50**	**SEM** ^b^	***P*** **-values**
MDA (nmol/ mgprot)	1.02^A^	0.69^B^	0.79^B^	0.71^B^	0.68^B^	0.025	<0.001
T-AOC (mmol/ gprot)	0.054^C^	0.067^A^	0.059^BC^	0.064^AB^	0.068^A^	0.001	<0.001
CAT (U/ mgprot)	7.65^D^	16.80^A^	10.28^C^	14.62^B^	17.54^A^	0.618	<0.001
T-SOD (U/ mgprot)	482.66^C^	515.91^A^	495.81^B^	512.83^A^	521.69^A^	2.787	<0.001

a *CON, basal diet without supplementation of trace minerals; IT 100, basal diet supplemented with Fe (60 ppm), Cu (10 ppm), Mn (80 ppm), and Zn (80 ppm); OT 20, basal diet+20% IT 100 trace minerals; OT 30, basal diet+30% IT 100 trace minerals; OT 50, basal diet+50% IT 100 trace minerals*.

b *SEM, Standard error of the mean*.

c *MDA, malondialdehyde; T-AOC, total antioxidant capacity; CAT, catalase; T-SOD, total superoxide dismutase*.

*Means within a row lacking a common superscript differ significantly (P < 0.05)*.

### Gene Expression Associated With Eggshell Quality

The expression of genes related to eggshell quality are shown in Table 6. IT100 treatment significantly upregulated *OC-116* expression; the same transcription levels were observed in OT30, whereas it significantly increased under OT50 treatment (*p* < 0.01). IT100 and OT50 treatments significantly upregulated *OCX-32* and *ALAS* (*p* < 0.01). Concerning *OC-17, OPN*, and *FECH*, IT100 had no significant effect, whereas OT50 treatment significantly increased their levels compared with CON (*p* < 0.01). No significant differences were detected between OT20 and CON groups concerning any gene analyzed. OT30 treatment upregulated *OPN* and *ALAS*, but only the effects on *ALAS* were similar to those of the IT100 group.

**Table 6:** Effects of inorganic and reduced substitution of organic trace minerals on gene expression of eggshell gland at the 8th week of the experiment with hen layer at the age of 67 weeks.

**Item**	**Treatment** ^a^						
	**CON**	**IT 100**	**OT 20**	**OT 30**	**OT 50**	**SEM** ^b^	***P*** **-values**
OC-116	0.73^D^	1.01^B^	0.82^CD^	0.92^BC^	1.25^A^	0.033	<0.001
OCX-32	0.65^C^	1.01^AB^	0.77^C^	0.88^BC^	1.18^A^	0.039	<0.001
OC-17	0.71^B^	1.04^AB^	0.72^B^	1.07^AB^	1.20^A^	0.050	0.002
OPN	0.92^C^	1.42^BC^	1.20^BC^	1.50^B^	4.31^A^	0.195	<0.001
ALAS	0.56^B^	1.00^A^	0.76^AB^	0.99^A^	1.04^A^	0.042	<0.001
FECH	0.83^B^	1.10^B^	1.24^AB^	1.30^AB^	1.66^A^	0.072	0.004

a *CON, basal diet without supplementation of trace minerals; IT 100, basal diet supplemented with Fe (60 ppm), Cu (10 ppm), Mn (80 ppm), and Zn (80 ppm); OT 20, basal diet+20% IT 100 trace minerals; OT 30, basal diet+30% IT 100 trace minerals; OT 50, basal diet+50% IT 100 trace minerals*.

b *SEM, Standard error of the mean*.

*Means within a row lacking a common superscript differ significantly (P < 0.05)*.

### Cecal Microbiota Populations

**Figure 1:**
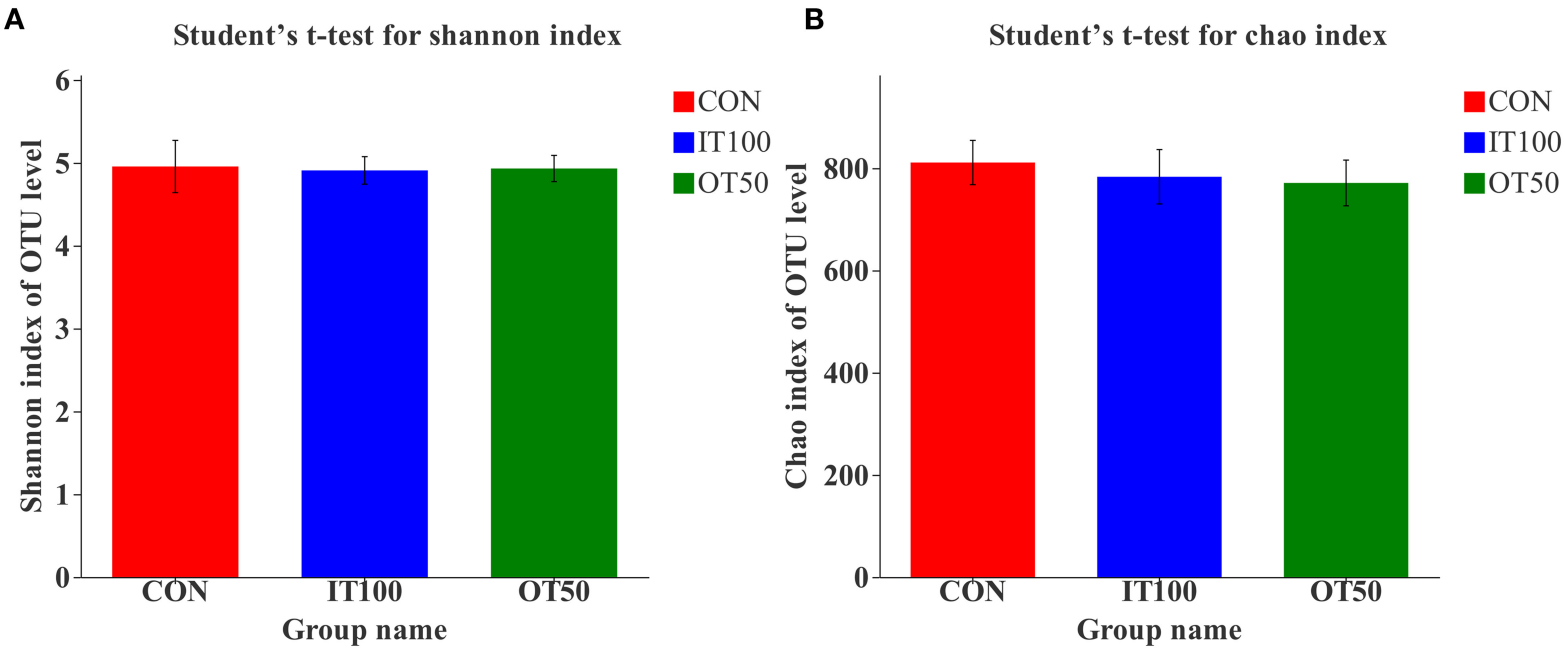
Effects of inorganic and low level of organic trace minerals on the α-diversity including Shannon index **(A)** and Chao index **(B)** of caecal microbiota at the eighth week of the experiment with hen layer at the age of 67 weeks (*n* = 6). CON: control treatment, basal diet without supplementation of trace minerals; IT 100: basal diet supplemented with Fe (60 ppm), Cu (10 ppm), Mn (80 ppm), and Zn (80 ppm) as sulfates; OT 50: basal diet+50% IT 100 trance minerals as amino acid chelates.

**Figure 2:**
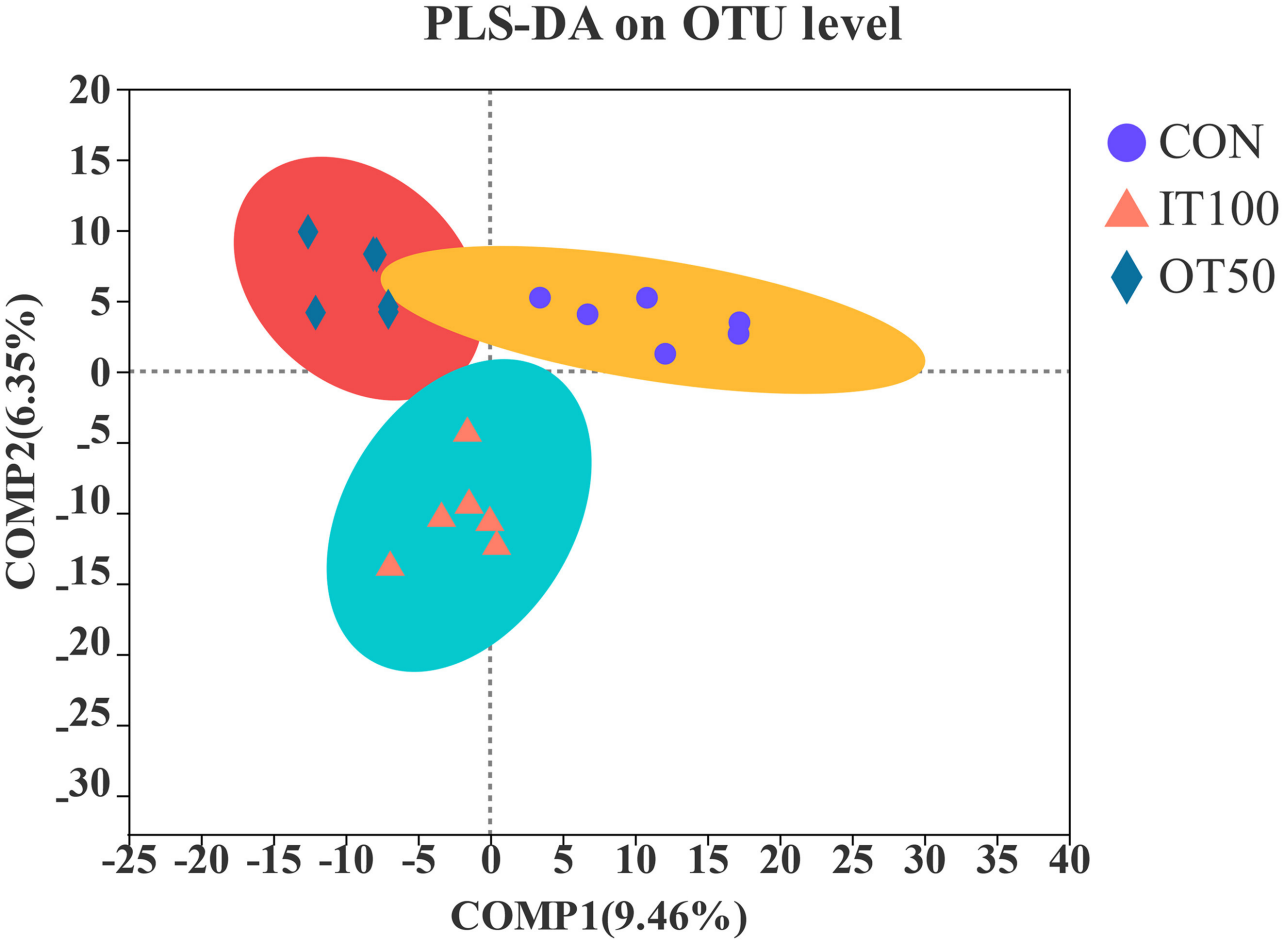
Effects of inorganic and low level of organic trace minerals on the caecal microbiota at the 8th week of the experiment with hen layer at the age of 67 weeks (*n* = 6). CON, control treatment, basal diet without supplementation of trace minerals; IT 100, basal diet supplemented with Fe (60 ppm), Cu (10 ppm), Mn (80 ppm), and Zn (80 ppm) as sulfates; OT 50, basal diet+50% IT 100 trance minerals as amino acid chelates.

**Figure 3:**
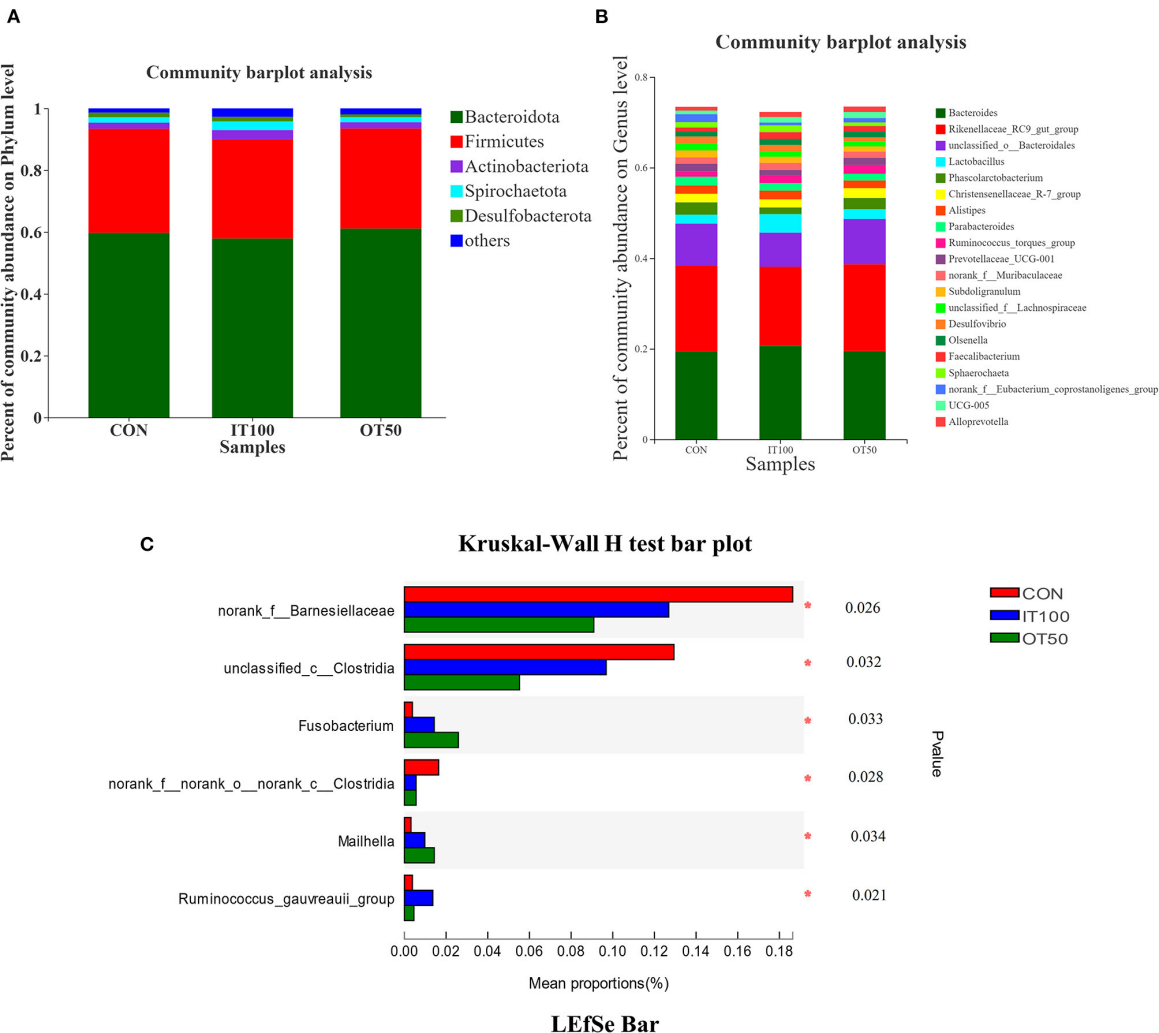
Effects of inorganic and low level of organic trace minerals on the caecal microbiota at the 8th week of the experiment with hen layer at the age of 67 weeks (*n* = 6). **(A,B)** No significant change was observed at phylum level and top 20 genus; **(C)** *Barnesiellaceae* and *Clostridia* were significantly decreased by supplement of inorganic or organic trace elements. CON, control treatment, basal diet without supplementation; IT 100, inorganic treatment 100%, basal diet with 100% Cu-Zn-Mn-Fe supplementation as sulfates; OT 50, organic treatment 50%, basal diet with 100% Cu-Zn-Mn-Fe supplementation as amino acid chelates. The asterisk symbol (*) means significantly different (*P* < 0.05).

**Figure 4:**
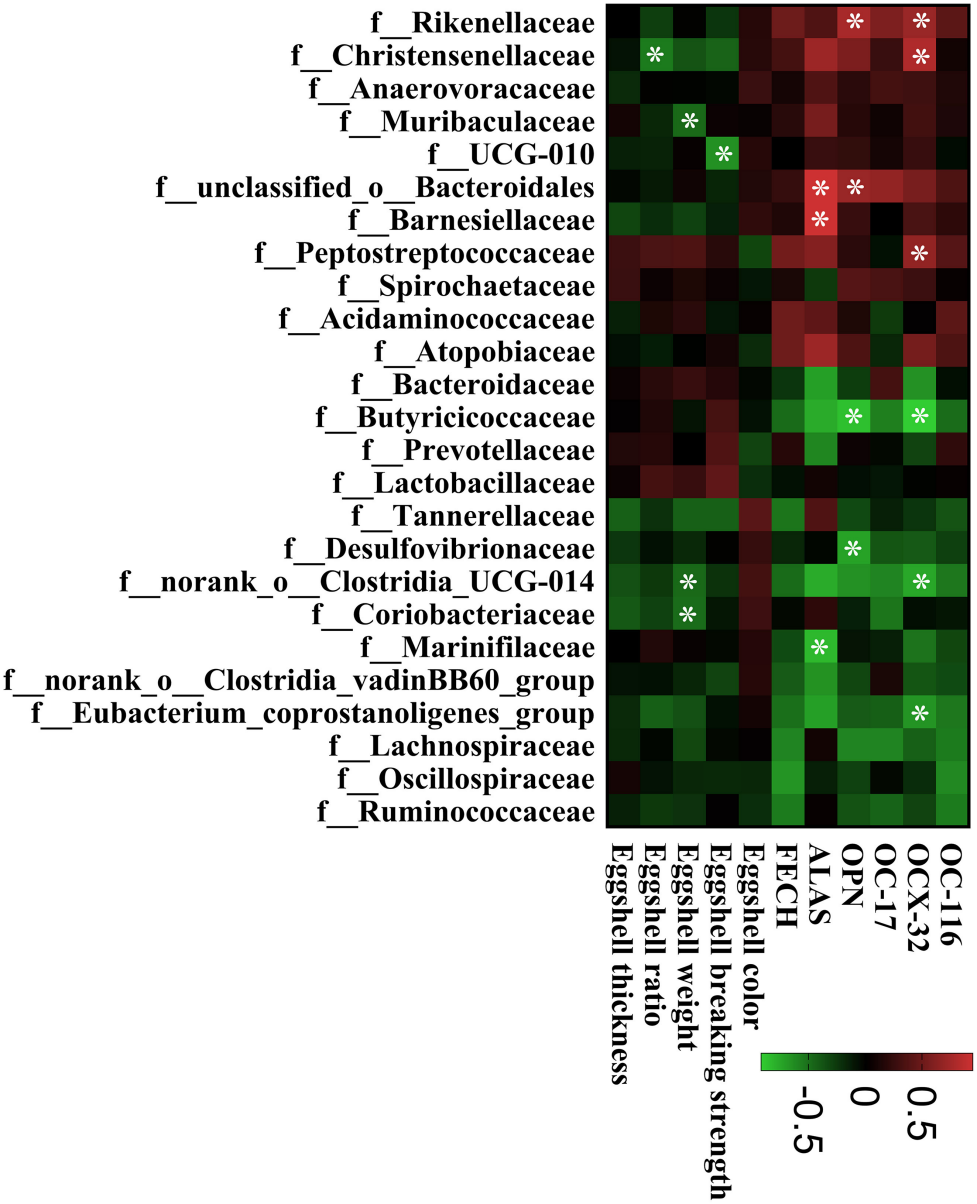
The correlation between the cecal bacterial communities and gene expression of eggshell gland, eggshell quality. The lattices were colored based on Spearman's rank correlation analysis. The red lattice indicates a positive correlation and the green lattice indicates a negative correlation. The asterisk symbol (*) means a significant correlation (*P* < 0.05).

The intestinal microbiota was examined in the cecum of CON, IT100, and OT50 groups. The α-diversity of the cecal microbiota measured by the Shannon and Chao indices was not significantly different among the treatment groups (Figure 1), whereas the β-diversity resultant from partial least-squares discriminant analysis differentiated the microbial community among the treatment groups (Figure 2). At the phylum level (relative abundance > 1.0%), *Bacteroidota* (57.9–61.1%), *Firmicutes* (32.1–33.7%), *Actinobacteriota* (2.0–2.9%), *Spirochaetota* (1.6–2.8%), and *Desulfobacterota* (0.9–1.5%) accounted for 98% of the total cecal microbiota; however, no significant differences were observed among the treatment group at the phylum level or for the top 20 genera (Figures 3A,B). Kruskal-Wallis H test further revealed that *Barnesiellaceae* and *Clostridia*, which had relative abundances > 0.1%, were significantly decreased in IT100 and OT50 (Figure 3C). Correlation analysis between the intestinal microbiota and the expression of genes related to the eggshell gland and quality is shown in Figure 4. *OCX-32* was positively correlated with *Rikenellaceae, Christensenellaceae*, and *Peptostreptococcaceae*, and negatively correlated with *Butyricicoccaceae, Eubacterium coprostanoligenes* group, and *Clostridia* UCG-014. *OPN* was positively correlated with *Rikenellaceae* and *Bacteroidales*, and negatively correlated with *Desulfovibrionaceae* and *Butyricicoccaceae*. *ALAS* was positively correlated with *Bacteroidales* and *Barnesiellaceae*, and negatively correlated with *Marinifilaceae*. As for the modulation of the eggshell quality, breaking strength, weight, and ratio were each negatively correlated with distinct microbiota. Eggshell breaking strength was negatively correlated with UCG-010; eggshell weight was negatively correlated with *Muribaculaceae, Clostridia* UCG-014, and *Coriobacteriaceae*; and the eggshell ratio was negatively correlated with *Christensenellaceae*. In contrast, the eggshell color and thickness were not associated with any cecal bacteria.

## Discussion

### Low Level of Organic Trace Minerals Showed the Same or Increased Deposition Effects as Inorganic Trace Minerals Treatment

Laying performance, average egg weight, feed intake, egg mass, feed conversion ratio and mortality rate were not significantly influenced by different sources of trace mineral or by reduced levels of organic minerals compared with CON group during the whole trial 1–8 week (15). Eggshell strength is a major concern, especially during the late production stages of laying hens. In this study our results showed that the laying performance was not significantly influenced by the supplementation of organic or inorganic complex trace minerals. Similar results were observed in broiler breeders and laying hens fed diets supplemented with organic trace minerals, and commercial level of inorganic trace minerals replaced by 50 or 70% complexed glycinates exhibited no significant effects on laying performance during the peak laying period (12, 16). Nevertheless, rapidly metabolizing tissues (i.e., the liver) respond rapidly to changes of intake in bioavailable Zn and have the highest deposition of Zn compared with that of jejunum and duodenum (17), thus the deposition of trace minerals was supposed to be measured to evaluated availability of organic trace minerals. Zn amino acid complexes increased the integrity of the intestinal epithelium by increasing the intestinal villus height, villus height/ crypt depth ratio in jejunum which enhance the absorption capacity of intestine (17). As deposition of Zn in the liver is highest compared with that of jejunum or duodenum and changed with different source and level of dietary Zn, the level of Zn in the liver was supposed to be an indicator of trace mineral availability. Source of mineral had positive effects on the concentration of liver Fe (18). Also, the antagonistic or synergistic relationship among Zn, Fe, and Cu in can be reflected by the liver concentrations of these elements (19). Therefore, hepatic concentration of these could be an indicator for the analysis of these trace minerals. We found that the content of Zn was increased in the liver of IT100 and OT50 compared with CON. Similar results were observed for the deposition of Cu in OT50 in the liver, whereas for the deposition of Mn and Fe, both OT30 and OT50 showed similar effects to IT100, indicating that deposition of organic Mn and Fe in the liver is more efficient than that of Cu and Zn. This finding can be partly explained by the interaction effects between trace minerals. The interactions between different trace minerals were observed that combination of supplementation of dietary Zn and Fe significantly decrease hepatic Cu levels with Cu was provided in low level in basal diet, and Cu supplementation significantly increase Fe concentration in the liver of hens fed Fe-supplemented diet (19). The higher concentrations of Cu, Mn, Zn, and Fe in the liver among OT 20-, OT 30-, and OT50-treated hens compared with CON group may be attributed to the increased amounts of dietary organic trace minerals in their diet. A previous study reported that the Zn content of eggshells, egg whites, and eggshell glands was similar in laying hens fed a diet containing 80 mg kg^−1^ Zn-Met and ZnSO_4_ (3). Moreover, Cu, Mn, Zn, and Fe deposition in the egg yolk was not influenced by supplementation of inorganic trace mineral. However, reduced supplementation of organic trace mineral (OT30 and OT50 treatments) significantly increased the Cu, Mn, Zn, and Fe deposition in the egg yolk, which also suggests that organic trace minerals have higher bioavailability than inorganic trace minerals. Deposition of minerals in the egg contents is related to the chemical form and amount of the mineral fed to the hen, with dietary minerals being transferred to the yolk *via* the ovary and to the albumen *via* the oviduct (20). Organic trace minerals in the form of HMTBA (2-hydroxy-4-[methylthio] butanoic acid) chelates (equal to 25% of the inorganic mineral treatment, 100-10-100 mg/kg Zn-Cu-Mn from sulfates) had similar mineral deposition in the entire egg contents compared with inorganic trace minerals (20). Compared with control group a basal diet supplemented with inorganic trace minerals (10-30-30 mg/kg Cu-Mn-Zn), replacement of Cu or Znas sulfates in the control by Cu or Zn in methionine hydroxy analog chelates form with the same proportion as control group, separately increased Cu or Zn concentration in the yolk during 39–52 weeks of age (21). The overall analysis of eggshell quality was shown in our previous study (15). Briefly, OT20, OT30, and especially OT50 treatments significantly increased the eggshell weight, ratio, thickness, and breaking strength compared with CON, and caused a darker eggshell color at week 8 of the experimental period Previous studies showed that Zn-methionine, as well as Cu, Mn, and Zn chelated with the hydroxy analog of methionine significantly increased eggshell thickness and strength compared with inorganic salt during the latter part of the laying cycle (22, 23).

### Low Level of Organic Trace Minerals Enhanced the Eggshell Strength *via* Modulation of Eggshell Gland Status

Eggshell glands are responsible for eggshell formation, which involves calcium carbonate synthesis and calcite crystal deposition facilitated by matrix proteins (i.e., osteopontin) (24). Zn, Cu and Mn is necessary for the structure and function of CuZnSOD and MnSOD, which catalyze the dismutation of superoxide anion into oxygen molecules (25). In a cadmium-induced disruption of eggshell gland of laying hen study, impaired shell biomineralization was accompanied with increased MDA, total nitric oxide synthase and decreased catalase, GPX in eggshell gland, which indicated that oxidative stress was one of the mechanisms of the toxic effects of cadmium on the eggshell gland of hens (24). Also, tamoxifen (the estrogen antagonist) helps to restore the antioxidant enzyme due to stress although it pauses egg laying, and blocks the estrogenic function as it is reducing the size of the shell gland, which suggested that estrogen directly influence the cell survival and egg-laying in the shell gland of hens mediated by its receptor alpha and anti-oxidant system (26). Thus, amelioration of oxidative stress may contribute to reducing the inhibition of estrogen inside the body, which increased the egg laying. In the present study, IT100 and OT50 enhanced the antioxidant status of the eggshell gland and might stabilize eggshell biomineralization by upregulating CAT, T-SOD, and T-AOC and downregulating MDA. The eggshell mineralization is the most efficient biological process of calcium mobilization and biomineralization (14). Deposition of organic and inorganic matter secreted by the shell gland makes up the different layers of eggshell (24). The organic matrix plays a key role in controlling crystallization and eggshell formation (27). The activation of genes involved in egg shell calcification process is time- and tissue-specific (28). Among the genes responsible for eggshell calcification, we found OC-116 and OCX-32 to be significantly upregulated in the uteri of laying hens. OC-116 regulates the organization of calcite crystals within the eggshell, and together with OCX-32, determines the elasticity and shell thickness and is involved in the termination of mineralization processes (28). OC-116 and OC-17 function as framework protein for calcite crystals deposition during mineralization process (28). OPN is synthesized and secreted by epithelial cells of the eggshell gland and is localized in the core of the unmineralized shell membrane fibers in the bases of mammillae and the outermost part of the palisade (27). It is an important protein for the formation of normal eggshells expressed uniformly by all epithelial cells facing the lumen. In contrast, pimpled or corrugated eggs are produced when OPN is expressed in the wrong section of the epithelium or not expressed in the eggshell gland, suggesting that OPN plays an important role in the formation of normal eggshells (27). A higher concentration of OPN leads to a smaller nanostructure size, which generally correlates with increased shell hardness (29). In our previous study, both IT 100 and OT 50 groups enhanced eggshell effective thickness and decreased mammillary thickness and mammillary knob width which contributed to the increase of eggshell breaking strength (15). In the present study, the expression of OC-116 was upregulated in IT100 and especially in OT20, OT30, and OT50. In addition, the expression levels of OCX-32, OC-17, and OPN were increased in OT50 compared with those in CON. Thus, enhancement of proteins in the eggshell matrix might contribute to the increase of eggshell strength and thickness.

Eggshell color is of great economic importance to the poultry industry. *ALAS* is a rate-limiting non-erythroid enzyme involved in the formation of the eggshell pigment protoporphyrin and is the first enzyme in heme synthesis (30). Inhibition of *ALAS* expression induced by nicarbazin in brown egg-laying hens resulted in less pigment protoporphyrin deposition into the eggshells (31). *FECH* is the last enzyme in heme synthesis and high levels convert more protoporphyrinogen into heme, reducing protoporphyrinogen deposition and leading to a light eggshell. Upregulation of *FECH* is detected in the spleen, but not in the uterus, of white eggshell hens compared with pink or brown eggshell hens (30, 32). In the present study, supplementation with organic or inorganic trace minerals increased the expression of *ALAS* in the eggshell gland; however, OT50 significantly increased the expression of *FECH*, which could lead to darker eggshells. Further research is needed to determine whether the high production of heme, protoporphyrin, or both, results in a darker eggshell.

### Low Level of Organic Trace Minerals Modulated the Intestinal Microbial Community

In the present study, we observed a remarkable differentiation of the microbial community among CON, IT100, and OT50 groups. Antibiotics were reported to decrease *Barnesiellaceae* and *Clostridia* abundance, while it downregulated pro-inflammatory cytokines, such as IL-1β and IL-8, in the cecum (33). Similarly, in our previous study, supplementation of both inorganic and 50% compound organic trace minerals were shown to reduce pro-inflammatory cytokines (IL-1β and IL-6), and increased IL-10, IgA, IgG, and IgM levels in the serum compared with control group (basal diet without additional trace minerals) (15). The diversity of gut microbiota is important for gastrointestinal homeostasis and host health (34). Enrichment of mucosa associated-microbiota including *Barnesiellaceae* at the genus level and *Clostridiales* order were observed in patients with primary sclerosing cholangitis which was associated with inflammatory bowel disease (35), indicating that decreased abundance of *Barnesiellaceae* and *Clostridiales* may be associated with the healthy intestine and increased absorption of trace minerals. In agreement with this finding, a previous study reported increased eggshell weight ratio and calcium retention, as well as a decreased abundance of intestinal *Clostridium* in laying hens fed a basal diet supplemented with *Bacillus subtilis* (36). Thus, modulation of intestinal bacteria can play an essential role in the improving eggshell quality and hen health.

In conclusion, our study suggests that low level of dietary complex organic trace minerals have maintained or enhanced the deposition of Cu, Mn, Zn, and Fe in the liver or egg yolk. Dietary organic trace mineral can improve the egg quality, enhance the antioxidant capacity and modulate genes involved in the formation of the organic matrix in the eggshell gland. In addition, dietary complex organic trace minerals reduced the abundance of *Barnesiellaceae* and *Clostridiales* in the cecum, improving the health of laying hens. Thus, reduced concentrations of complex organic trace minerals may be a practicable candidate for reducing the use of inorganic trace minerals in laying hens during the late production stage.

## Data Availability Statement

The original contributions presented in the study are publicly available. This data can be found here: https://www.ncbi.nlm.nih.gov/bioproject/PRJNA738271 / accesion number PRJNA738271.

## Ethics Statement

The animal study was reviewed and approved by the Animal Health and Care Committee of the Shanxi Agricultural University.

## Author Contributions

YD, KZ, and JL contributed to conception and design of the study. YD and KZ organized the database. MH and CL performed the statistical analysis. YD and ZM wrote the first draft of the manuscript. YD and JL wrote sections of the manuscript. All authors contributed to manuscript revision, read, and approved the submitted version.

## Funding

This work was supported by the Fund Program for the Scientific Activities of Selected Returned Overseas Professionals in Shanxi Province (20220016), Shanxi Agricultural University Science and Technology Innovation Fund under Grant (2021BQ03), Shanxi Province Outstanding Doctor Award Fund (SXBYKY2021033), Science and Technology Innovation Project of Shanxi Provincial Department of Education (2021L143), and 1331 Project Key Disciplines of Animal Science Shanxi Province (J202011313). The funding agencies had no role in the collection, analysis, and interpretation of data, in the writing of the report, or in the decision to submit the article for publication.

## Conflict of Interest

The authors declare that the research was conducted in the absence of any commercial or financial relationships that could be construed as a potential conflict of interest.

## Publisher's Note

All claims expressed in this article are solely those of the authors and do not necessarily represent those of their affiliated organizations, or those of the publisher, the editors and the reviewers. Any product that may be evaluated in this article, or claim that may be made by its manufacturer, is not guaranteed or endorsed by the publisher.
